# Pharmacological modulation of autophagy enhances Newcastle disease virus-mediated oncolysis in drug-resistant lung cancer cells

**DOI:** 10.1186/1471-2407-14-551

**Published:** 2014-07-30

**Authors:** Ke Jiang, Yingchun Li, Qiumin Zhu, Jiansheng Xu, Yupeng Wang, Wuguo Deng, Quentin Liu, Guirong Zhang, Songshu Meng

**Affiliations:** Institute of Cancer Stem Cell, Dalian Medical University Cancer Center, 9 Lvshun Road South, Dalian, 116044 China; Biotherapy Research Center, Liaoning Cancer Hospital & Institute, 44 Xiaoheyan Road, Shenyang, 110042 China; Dalian Central Hospital, 826 Southwest Road, Dalian, 116033 China; Ministry of Education Key Lab for Avian Preventive Medicine, College of Veterinary Medicine, Yangzhou University, 48 Wenhuidong Road, Yangzhou, 225009 China

**Keywords:** Newcastle disease virus, Autophagy, Apoptosis, Drug resistance, Lung cancer, Virotherapy

## Abstract

**Background:**

Oncolytic viruses represent a promising therapy against cancers with acquired drug resistance. However, low efficacy limits its clinical application. The objective of this study is to investigate whether pharmacologically modulating autophagy could enhance oncolytic Newcastle disease virus (NDV) strain NDV/FMW virotherapy of drug-resistant lung cancer cells.

**Methods:**

The effect of NDV/FMW infection on autophagy machinery in A549 lung cancer cell lines resistant to cisplatin (A549/DDP) or paclitaxel (A549/PTX) was investigated by detection of GFP-microtubule-associated protein 1 light chain 3 (GFP-LC3) puncta, formation of double-membrane vesicles and conversion of the nonlipidated form of LC3 (LC3-I) to the phosphatidylethanolamine-conjugated form (LC3-II). The effects of autophagy inhibitor chloroquine (CQ) and autophagy inducer rapamycin on NDV/FMW-mediated antitumor activity were evaluated both in culture cells and in mice bearing drug-resistant lung cancer cells.

**Results:**

We show that NDV/FMW triggers autophagy in A549/PTX cells via dampening the class I PI3K/Akt/mTOR/p70S6K pathway, which inhibits autophagy. On the contrary, NDV/FMW infection attenuates the autophagic process in A549/DDP cells through the activation of the negative regulatory pathway. Furthermore, combination with CQ or knockdown of ATG5 significantly enhances NDV/FMW-mediated antitumor effects on A549/DDP cells, while the oncolytic efficacy of NDV/FMW in A549/PTX cells is significantly improved by rapamycin. Interestingly, autophagy modulation does not increase virus progeny in these drug resistant cells. Importantly, CQ or rapamycin significantly potentiates NDV/FMW oncolytic activity in mice bearing A549/DDP or A549/PTX cells respectively.

**Conclusions:**

These results demonstrate that combination treatment with autophagy modulators is an effective strategy to augment the therapeutic activity of NDV/FMW against drug-resistant lung cancers.

## Background

Acquired drug resistance to first-line chemotherapeutics, such as cisplatin and paclitaxel, is a major factor contributing to chemotherapy failure in non-small cell lung cancer (NSCLC) patients [1, 2]. Oncolytic viruses (OVs) are emerging as new cancer therapeutic approaches with great potential for the treatment of drug-resistant lung cancers [3]. We previously reported that the oncolytic Newcastle disease virus (NDV) induces apoptosis in cisplatin-resistant A549 (A549/DDP) cells *in vitro* and *in vivo* [4]. NDV is an avian paramyxovirus that selectively replicates in a variety of tumor cells but not in normal human cells [5]. NDV strains such as LaSota, Ulster [6], 73-T [7], NDV/FMW [8, 9], and NDV- HUJ [10, 11] have displayed oncolytic effects in lung cancer cells. Notably, in addition to triggering apoptosis in chemo-resistant malignant primary melanoma [12], oncolytic NDV induces efficient oncolysis in human lung adenocarcinoma A549 cells over-expressing Bcl-xL, a known anti-apoptotic protein [13]. These studies and studies from our lab indicate a potential role of oncolytic NDV in the treatment of drug-resistant lung cancers. However, it remains a challenge to improve the efficacy of NDV in drug-resistant NSCLC cells in preclinical and clinical tests.

Oncolytic NDV is known to trigger apoptosis pathways in infected tumor cells [4, 8, 10, 14–16]. In addition to targeting the cellular apoptosis machinery, we recently reported that oncolytic NDV induces autophagy in U251 human glioma cells to promote virus production [17], suggesting that autophagy may be involved in NDV-induced oncolysis. Autophagy is a conserved homeostatic mechanism of lysosomal degradation [18]. The hallmark of autophagy is a double-membraned autophagosome that engulfs long-lived cytoplasmic macromolecules and damaged organelles [19]. Autophagy is mainly modulated by the mTOR (mammalian target of rapamycin) and PI3K (phosphatidylinositol 3-kinase) pathways, which are class I (inhibitory to autophagy) and class III (necessary for the execution of autophagy) modulators [20, 21]. Accumulating evidence reveals that OVs interact with the autophagy machinery in infected tumor cells, and autophagy plays a role in OV-mediated cancer cell death [22–24]. Of note, a number of studies reported that the pharmacological modulation of autophagy augments the anti-tumor effects of OVs, such as the oncolytic adenovirus OBP-405 in combination with the autophagy inducers temozolomide, rapamycin and RAD001 in glioma cells [25], *dl*922-947 in combination with the autophagy inhibitor chloroquine (CQ) in glioma cells [26], Ad-cycE with rapamycin in lung cancer cells [27]. In addition, autophagy plays critical roles in both innate and adaptive immuninity. It has been shown that autophagy enhances tumor immunogenicity via releasing damage-associated molecular pattern (DAMP) molecules by dying cells with autophagy and promoting antigen cross presentation from cancer cells by DCs to naive T cells [28, 29]. Since OV infections can interact with the cellular autophagy machinery, OV in combination with an autophagy modulator would enhance the antitumor immune responses, thereby improving OV-mediated efficacy [29–31]. Together, data from these studies strongly indicate that targeting autophagy may be utilized as a novel strategy for enhancing the oncolytic virotherapy of cancers.

The objective of this study was to investigate whether pharmacologically targeting autophagy could enhance NDV virotherapy in drug-resistant lung cancer cells. We first dissected the interaction between NDV and the cellular autophagy machinery in cisplatin- and paclitaxel-resistant A549 lung cancer cells and further demonstrated that the modulation of autophagy with rapamycin or CQ enhances the NDV-mediated anti-tumor effects on drug-resistant A549 cells *in vitro* and *in vivo*. Therefore, our results suggest that combination with chemotherapeutic agents that modulate autophagy may be a potential strategy to optimize the clinical efficacy of oncolytic NDV.

## Methods

### Cell lines, mice and virus preparation

A549 human lung cancer cell line and chicken embryo fibroblast cell line DF1 was purchased from American Type Culture Collection (ATCC) and cultured at 37°C and 5% CO_2_ in DMEM supplemented with 10% fetal bovine serum (FBS). Cisplatin-resistant A549 (A549/DDP) cells [4] were cultured in DMEM containing 2 μg/mL cisplatin (Sigma) to maintain resistance. An A549-derived paclitaxel-resistant sub-line, A549/PTX, was kindly provided by Dr. Sang Kook Lee (Seoul National University) and cultured in RPMI 1640 containing 100 ng/mL paclitaxel (Sigma) to maintain resistance[32]. The cells were cultured in complete media without cisplatin or paclitaxel for 3 days before performing experiments. The NDV strain NDV/FMW, which has been previously shown to be oncolytic in A549/DDP and parental cells [4, 8], was used throughout the study. Virus passaging, propagation, and titration were performed as previously described, and virus titer was expressed as log_10_ 50% tissue culture infective dose (TCID_50_) [8]. BALB/c nude mice (female, 4–6 weeks old) were purchased from the Experimental Animal Center of Dalian Medical University (Dalian, China) and all procedures involving animals and their care complied with the China National Institutes of Healthy Guidelines for the Care and Use of Laboratory Animals. Ethical approval for the study was granted by the Ethics Committee of Dalian Medical University.

### Antibodies and reagents

The monoclonal anti-Beclin-1 antibody and high-mobility group box1(HMGB1) were purchased from Santa Cruz. The polyclonal rabbit anti-microtubule-associated protein 1A/1B-light chain 3 (LC3) and a monoclonal antibody against *β*-Actin were obtained from Sigma. The following antibodies were purchased from Cell Signaling Technology: cleaved caspase-3 and phospho-specific antibodies to mTOR (Ser2448), Akt (Ser473) and p70 ribosomal protein S6 kinase (S6K) (Thr389), along with total antibodies directed against mTOR, Akt, and p70S6K. Rapamycin and chloroquine (CQ) were purchased from Sigma.

### Virus infection

A549/DDP, A549/PTX, and parental A549 cells were infected with NDV/FMW at a multiplicity of infection (MOI) of 10, or they were sham-infected with phosphate-buffered saline (PBS), at 37°C for 1 h in serum-free DMEM. The cells were washed three times with PBS and incubated at 37°C in reduced serum (1% FBS)-containing media. For the pharmacological modulation of autophagy, cells were treated with rapamycin (100 nM) or CQ (5 μM) for 30 min prior to virus infection. Subsequently, the cells were infected with NDV/FMW in the presence or absence of various compounds for 1 h and then cultured in fresh DMEM or RPMI 1640 containing rapamycin or CQ for the indicated times. For experiments that involved the determination of virus yield, tumor cells were infected with NDV/FMW at an MOI of 0.01, and multi-step viral growth curves were measured as previously described [8].

### Cell transfection and fluorescence microscopy

Tumor cells were transfected with a plasmid expressing green fluorescent protein (GFP)-LC3 using Lipofectamine 2000 according to the manufacturer’s instructions. Dot formation by GFP-LC3 was detected with a fluorescence microscope (BX50, Olympus) following drug treatment and/or NDV/FMW infection. Transfected cells with five or more puncta were considered to have accumulated autophagosomes. A total of 100 transfected cells were examined per well in triplicate from three independent experiments.

### RNA interference

RNA interference was used to knock down ATG5, a key gene for autophage formation. Two siRNA oligonucleotides were used: ① 5'-TGA TAT AGC GTG AAA CAA G-3' [33]; ② 5'-CAA CTT GTT TCA CGC TAT A--3' [34]. Transfection of siRNA was performed as described previously [17, 35]. A scrambled siRNA was used as a negative control. The silencing efficiency was detected by immunoblot. At 48 h after transfection, cells were infected with NDV/FMW at an MOI of 10 for various times.

### Transmission electron microscopy analysis

Standard transmission electron microscopy (TEM) was performed as previously described [17]. Briefly, 24 h after NDV/FMW infection, the cells were fixed and embedded. Thin sections (90 nm) were examined at 80 kV with a JEOL 1200EX transmission electron microscope. Approximately 15 cells were counted, and autophagosomes were defined as double-membrane vacuoles measuring 0.5 or 2.0 μm.

### Cell proliferation assay

Tumor cells were seeded into 96-well plates, and cell growth was measured daily by the MTT assay as previously described [8]. The experiments were repeated three times.

### Flow cytometric analysis of apoptosis

Apoptosis was quantified using flow cytometry as previously described [8]. Briefly, tumor cells were seeded at 1 × 10^5^ cells/dish in 60-mm dishes and treated with NDV/FMW at an MOI of 10. Floating cells and cell pellets were prepared for the annexin V-fluorescein isothiocyanate (FITC) and propidium iodide (PI) double-staining procedure. The cell population in the lower right quadrant (PI-negative, annexin V-positive) corresponds to apoptotic cells. The data was determined in three independent experiments.

### Immunoblot assay

Immunoblot (IB) assays were performed as described previously [36]. Densitometry analysis of the specific protein expression was performed using a calibrated GS-670 densitometer. All IB experiments were performed in duplicate.

### Animal experiments

Nude mice were subcutaneously inoculated in the flank with A549/DDP and A549/PTX cells (5 × 10^6^ cells in 100 μL PBS/mouse) to induce tumor development. When tumors reached an average volume of 200 mm^3^, tumor-bearing mice were intratumorally inoculated with NDV/FMW. Mice were randomly divided into four groups (six mice per group): (a) vehicle treatment, (b) intraperitoneal (i.p.) treatment with rapamycin (5 mg/kg) or CQ (45 mg/kg) alone three times a week, (c) intratumoral administration with NDV/FMW (1 × 10^7^ TCID_50_ per dose) three times a week, and (d) NDV/FMW treatment in combination with CQ or rapamycin (same dose as described previously) administered 1 d prior to virus injection. One week after treatment, two mice (of six) were sacrificed, and tumor sections (5 μm) were subjected to either hematoxylin–eosin (H&E) staining or terminal deoxynucleotidyl transferase dUTP nick end labeling (TUNEL) assay as previously described [4, 9]. TUNEL-positive (brown staining) cells were characterized as apoptotic cells, and 10 randomly selected microscopic fields in each group were examined to calculate the ratio of TUNEL-positive cells. Tumor tissue samples from two different mice (of six) from each treatment group were subjected to immunoblot analysis to evaluate cleaved caspase-3 levels or LC3II abundance. Excised tumors from the other two animals (of six) were subjected to virus isolation.

For the *in vivo* oncolysis study, 10 mice were included in each treatment group, and the four mouse groups were treated as described above for two weeks. At five-day intervals, mice were examined for tumor growth or survival. Tumor diameter was measured with a caliper, and tumor volume was calculated based on the following formula: volume = (greatest diameter) × (smallest diameter) ^2^/2. The experiment was terminated when tumors reached 1 cm^3^ in volume and/or symptomatic tumor ulceration occurred, and the surviving mice were sacrificed under anesthesia.

### Statistical analysis

Comparisons of data for all groups in the viral propagation and cytotoxicity assays were first performed using one-way analysis of variance (ANOVA). Multiple comparisons between treatment groups and controls were evaluated using Dunnett’s least significant difference (LSD) test. To assess *in vivo* oncolytic effects, statistical significance between groups was calculated using the LSD test in SPSS 17.0 software (SPSS Inc., Chicago, IL, USA). A p < 0.05 was considered statistically significant.

## Results

NDV/FMW induces autophagosome formation in paclitaxel-resistant A549 cells but attenuates the autophagic process in cisplatin-resistant A549 cells.

**Figure 1:**
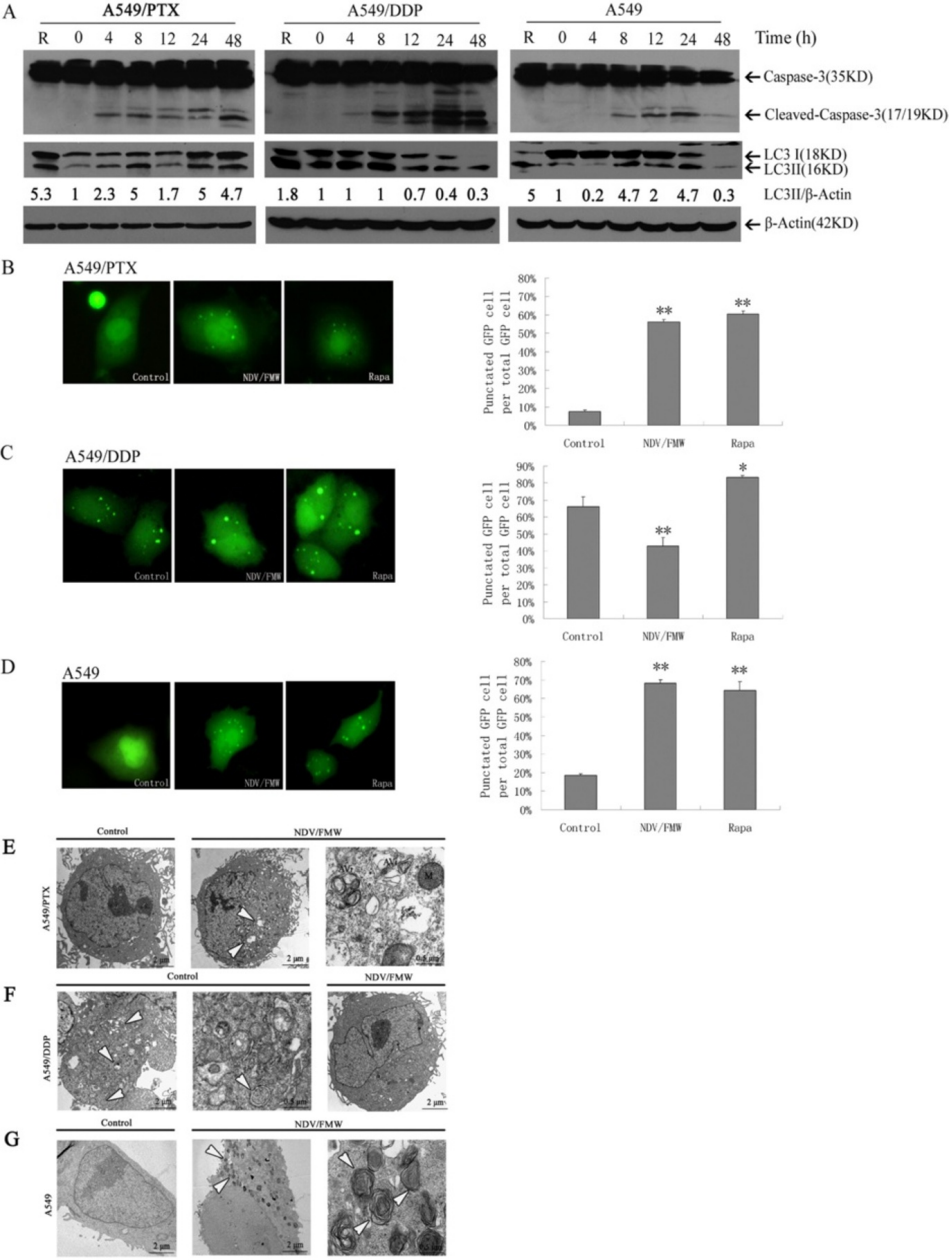
**Oncolytic NDV/FMW induces apoptosis and modulates autophagy in drug-resistant lung cancer cells.** Paclitaxel-resistant A549 (A549/PTX) and cisplatin-resistant A549 (A549/DDP) and parental cells were infected with NDV/FMW at a multiplicity of infection (MOI) of 10, and at the indicated time points. **(A)** Activation of caspase-3 and LC3I to LC3II conversion were analyzed by immunoblot (IB) assay, using *β*-Actin as a loading control. R stands for rapamycin, an autophagy inducer used as the positive control. Densitometry was performed for quantification, and the ratios of LC3II to *β*-Actin are presented below the blots. The results shown are representative of two separate experiments. **(B-D)** Drug-reisistant A549 and parental cells were transfected with GFP-LC3, followed by NDV/FMW infection for 24 h. The pictures show mock-infected cells, cells treated with rapamycin for 24 h as a positive control. The number of cells with punctated GFP-LC3 is displayed as a histogram. *p < 0.05;**p < 0.01. **(E-G)** Transmission electron microscopy analysis of cells infected with NDV/FMW for 24 h. **(E)** NDV/FMW-infected A549/PTX cells displayed more vacuolated (indicated by the arrows) than control (uninfected cells), the enlarged image showed initial autophagosomes (AVi) and a swollen mitochondrion (M) in infected A549/PTX cells. **(F)** Uninfected-A549/DDP cells showed disappearance of most organelles, the two limiting membranes of the autophagosome are visible in enlarged image (indicated by the arrows), and infected A549/DDP cells showed normal distribution of organelles and few autophagic structures. **(G)** Infected A549 cells showed highly autophagosome (indicated by the arrows) rather than uninfected A549 cells, clearer autophagosome showed in the enlarged image. Data shown are representative of three independent experiments.

We previously reported that oncolytic NDV induces apoptosis in cisplatin-resistant A549 (A549/DDP) and parental cells [4, 8]. Here, we show that marked caspase-3 cleavage was detected in paclitaxel-resistant A549 (A549/PTX) cells upon NDV/FMW infection (Figure 1, left panel), indicating that NDV/FMW infection induces apoptosis in paclitaxel-resistant A549 cells. Our recent study revealed that NDV infection activated autophagy in cancer cells [17]; however, the significance related to NDV-mediated oncolysis has not been elucidated. To investigate whether NDV/FMW interacts with the autophagy machinery in drug-resistant A549 and parental cells, we first examined the conversion of LC3I (cytosolic form) to LC3II (autophagosome-bound lipidated form), a hallmark of autophagy [37]. Consistent with a previous report [38], A549/DDP cells displayed high basal levels of LC3II, which remained unchanged upon NDV/FMW infection at 4 and 8 hours post-infection (hpi) (Figure 1A, middle panel). However, the LC3II abundance was markedly diminished at 12 and 24 hpi (Figure 1A, middle panel), suggesting that NDV infection reduces LC3 conversion in the late stage of viral infection. In contrast, increased LC3II abundance was detected in A549/PTX and parental cells after NDV/FMW infection (Figure 1A, left and right panels), indicating that NDV infection induces LC3 conversion in these cells.

To determine whether NDV/FMW perturbs autophagosome formation in drug-resistant A549 cells, we detected GFP-LC3 dot formation, which is generally regarded as an autophagosome [37]. A549/DDP, A549/PTX, and parental cells were transfected with GFP-LC3 and then mock-infected or infected with NDV/FMW at an MOI of 10. As shown in Figures 1B and D, the GFP-LC3 redistribution into discrete dots was significantly increased in NDV/FMW -infected A549/PTX (**p < 0.01) and parental (**p < 0.01) cells at 24 hpi, while a diffuse cytoplasmic distribution of fluorescence was observed in mock-treated A549/PTX and parental cells. Interestingly, marked punctated GFP-LC3 accumulation was observed in mock-infected A549/DDP cells (Figure 1C), suggesting a high basal level of autophagy. However, upon NDV/FMW infection, the number of A549/DDP cells with punctated GFP-LC3 was significantly diminished compared to basal levels (Figure 1C, **p < 0.01). Control cells treated with the autophagy inducer rapamycin exhibited typical GFP-LC3 dot formation. In addition, TEM-based ultrastructural analysis of the formation of double-membrane vesicles (autophagosomes) confirmed the above findings (Figures 1E, F, and G). Therefore, these results indicate that NDV/FMW induces autophagosome formation in A549/PTX and parental cells, whereas it inhibits the autophagic process in A549/DDP cells.

### NDV/FMW infection perturbs autophagic signaling pathways in drug-resistant A549 cells

**Figure 2:**
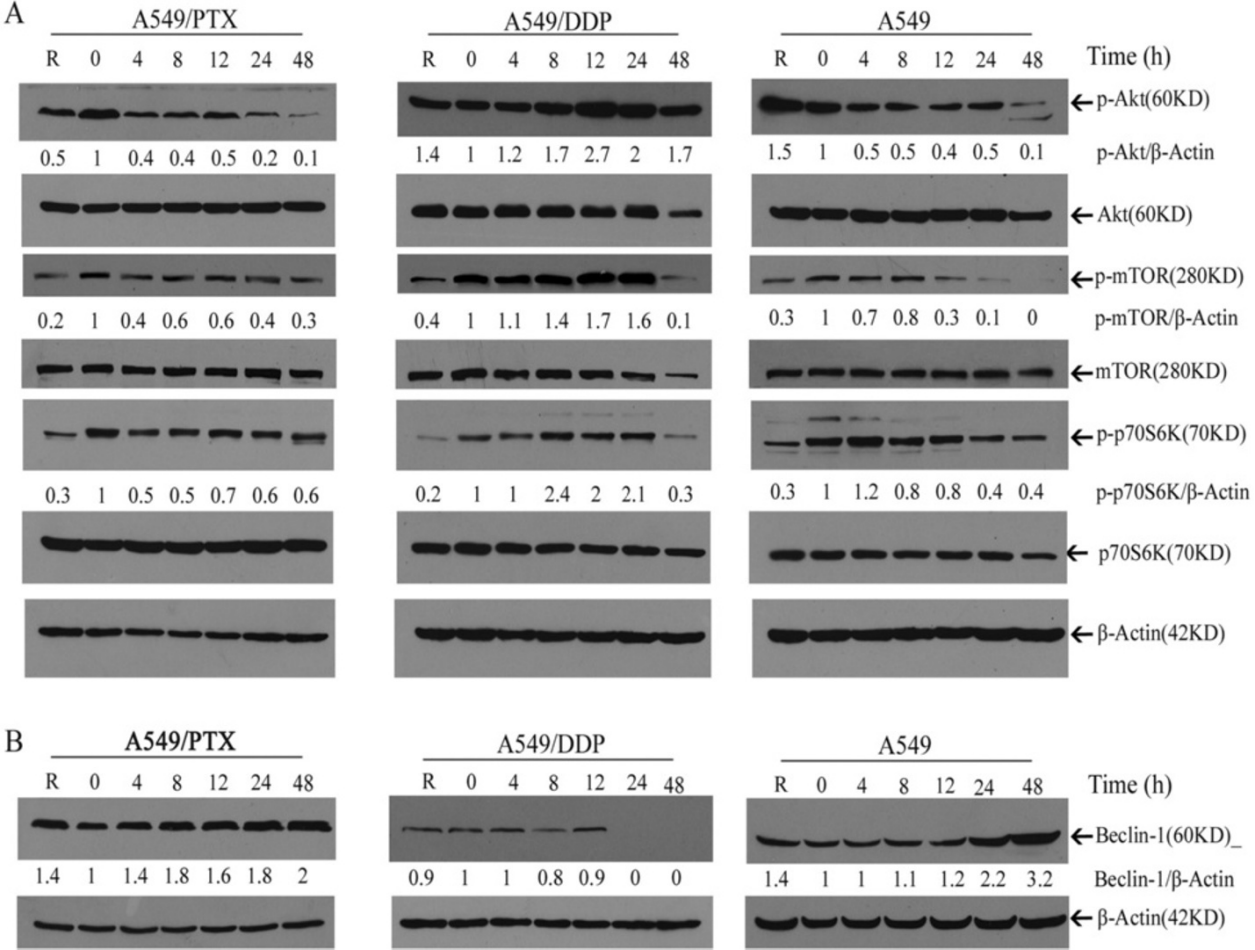
**Autophagic signaling pathways are regulated in response to NDV/FMW infection in drug-resistant lung cancer cells.** A549/DDP and A549/PTX as well as parental cells were infected with NDV/FMW at an MOI of 10 at the indicated time points. Expression of beclin-1 **(B)**, total and phosphorylated (p-) Akt, mTOR and p70S6K **(A)** was analyzed by immunoblot, using *β*-Actin as a loading control. R stands for rapamycin, an autophagy inducer inhibiting mTOR phosphorylation. The ratios of phosphorylated protein to *β*-Actin are presented below the blots. Results shown are representative of three independent experiments.

To elucidate the underlying mechanisms of the different patterns of autophagy modulation in various drug-resistant A549 cells upon NDV/FMW infection, we examined the class I PI3K/Akt/mTOR/p70S6K and class III PI3K/Beclin-1 pathways, which negatively (the former) or positively (the latter) regulate autophagosome formation [20, 21]. As shown in Figure 2A (left and right panels), NDV/FMW infection reduced the phosphorylation levels of Akt in A549/PTX and A549 cells in a time-dependent manner, indicating inhibition of the negative regulatory pathway in autophagy. In line with our previous work [4], we observed a time-dependent increase in Akt phosphorylation in A549/DDP cells upon NDV/FMW infection (Figure 2A, middle panel), indicating activation of the negative regulatory pathway in autophagy. Accordingly, we detected increased mTOR and p70S6K phosphorylation in NDV/FMW -infected A549/DDP cells (Figure 2A, middle panel) and marked reductions in mTOR and p70S6K phosphorylation in A549/PTX and parental cells (Figure 2A, left and right panels). No change was detected in the levels of total Akt, mTOR, and p70S6K. Together, these observations indicate that the class I PI3K/Akt/mTOR/p70S6K signaling pathway contribute to the interaction between the NDV and autophagy machinery in drug-resistant A549 and parental cells.

Beclin-1 forms a complex with class III PI3K and plays an essential role in controlling the first steps of autophagy commitment [39]. We found that beclin-1 expression was up-regulated in a time-dependent manner in NDV-infected A549/PTX and parental cells (Figure 2B, left and right panels), suggesting that beclin-1 may participate in the induction of autophagosome formation in these cells during NDV/FMW infection. Upon NDV/FMW infection, the expression of beclin-1 in A549/DDP cells was nearly unchanged from 4 to 12 hpi and was completely diminished at 24 hpi (Figure 2B, middle panel), suggesting that NDV/FMW infection decreases beclin-1 expression in the late stage of infection. Therefore, these data indicate that the class III PI3K/Beclin-1 pathway may be involved in the interplay between NDV/FMW and the cellular autophagy machinery in drug-resistant A549 and parental cells.

### Pharmacological modulation of autophagy enhances NDV/FMW-induced cytotoxicity

**Figure 3:**
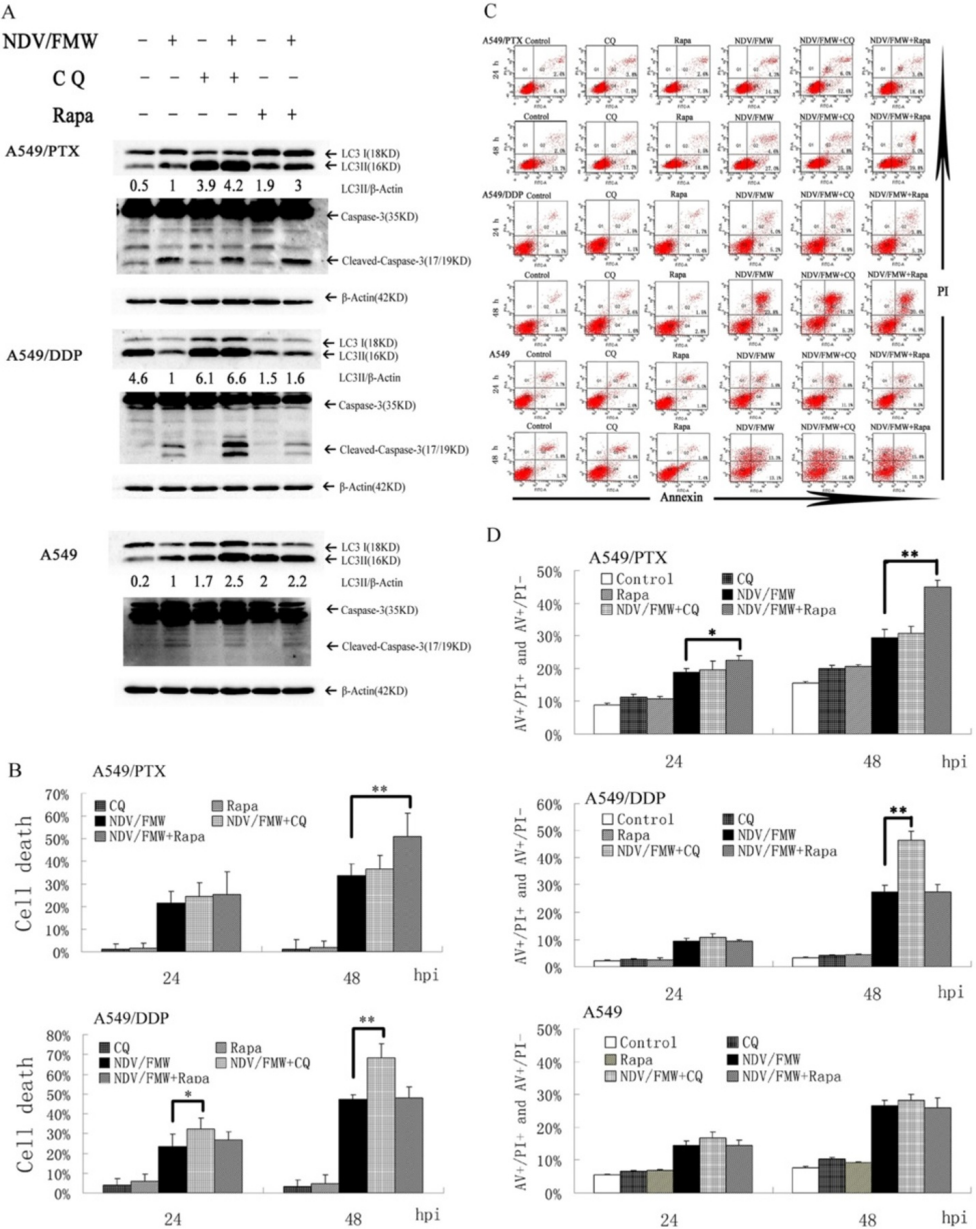
**Pharmacological autophagy modulators enhance NDV/FMW-induced cytotoxicity.** A549/DDP and A549/PTX as well as parental cells were treated with chloroquine (CQ, or rapamycin or vehicle for 30 min and infected with NDV/FMW (MOI = 10) or mock-infected for various times. **(A)** LC3II conversion and caspase-3 cleavage at 24 h post-infection were monitored by immunoblot analysis. The ratios of LC3II to *β*-Actin are presented below the blots. CQ (7.5 μM) and rapamycin (125 nM) were used. **(B)** Cell viability at 24 and 48 h post-infection (hpi) was determined by the MTT assay. CQ (5 μM) and rapamycin (100 nM) were used. Data presented are mean ± SD calculated from three independent experiments (*p < 0.05; **p < 0.01). **(C** **and** **D)** Cells at 24 and 48 h post-infection were double-stained with annexin V-FITC and propidium iodide (PI), apoptosis was assessed by FACS analysis. CQ (5 μM) and rapamycin (100 nM) were used. Bar graph summarized the percentage of apoptotic cells from three independent experiments (*p < 0.05, **p < 0.01).

We sought to elucidate whether the efficacy of the oncolytic NDV/FMW virotherapy of drug-resistant lung cancer cells could be enhanced by combination with autophagy modulators. To this end, we used the autophagy inducer rapamycin and the autophagy inhibitor CQ because these two compounds and their analogs, including RAD001 and hydroxychloroquine, have been widely employed to potentiate the anti-tumor effects of several oncolytic viruses in preclinical settings. Importantly, these compounds have been approved for use in clinical trials [25, 40–42]. Rapamycin selectively targets mTOR to stimulate autophagy, while CQ is known to disrupt autophagosome-lysosome fusion, leading to the accumulation of autophagic vacuoles, as demonstrated by a marked accumulation of LC3II [43, 44]. Importantly, CQ has been used to overcome resistance of lung carcinoma cells to different chemotheraputics such as the dual PI3K/mTOR inhibitor PI103 and crizotinib [45, 46], while rapamycin has been administrated in a phase I trial of patients with advanced non-small cell lung cancer [47]. The two compounds had no effect on cell viability at the concentrations used in our preliminary trial. As seen in Figure 3A, the pre-addition of either rapamycin or CQ to drug-resistant A549 and parental cells resulted in enhanced LC3II accumulation upon NDV/FMW infection compared with control infection. Together, these results indicate an enhanced induction of autophagy by rapamycin and inhibition of autophagosome-lysosome fusion by CQ in infected cells.

We then examined whether the pharmacological modulation of autophagy had an effect on NDV/FMW-mediated cytotoxicity. NDV/FMW-mediated cell death in rapamycin-treated A549/PTX and CQ-treated A549/DDP cells was significantly augmented as determined by the MTT assay (Figure 3B). FACS analysis demonstrated that the pre-addition of rapamycin rather than CQ to A549/PTX cells significantly increased the number of apoptotic cells upon NDV/FMW infection compared with NDV/FMW infection alone (Figure 3C and 3D, *p < 0.05; **p < 0.01), supporting by the observation that treatment with rapamycin but not CQ enhanced the cleavage of caspase 3 in NDV/FMW-infected A549/PTX cells compared with virus alone (Figure 3A). Together, these results suggest that autophagy may function as a death mechanism in NDV/FMW-infected A549/PTX cells, and augmenting the autophagic response with rapamycin increases viral cytotoxicity. Conversely, CQ, but not rapamycin, increased the activation of caspase-3 in NDV/FMW-infected A549/DDP cells compared with NDV/FMW infection alone (Figure 3A), while treatment with CQ rather than rapamycin significantly increased apoptosis and necrosis or a late necrosis consecutive to apoptosis in NDV/FMW-infected A549/DDP cells, as demonstrated by the FACS analysis (Figure 3C and 3D, **p <0.01). These data indicate that autophagy may act as a survival mechanism in NDV/FMW-infected A549/DDP cells, and the attenuation of the autophagic response enhances viral oncolysis. Interestingly, treatment with neither rapamycin nor CQ exerted an effect on the cleavage of caspase-3 in NDV/FMW-infected A549 cells (Figure 3A). As expected, no significant change in the number of apoptotic cells was detected in rapamycin- or CQ-treated A549 cells upon NDV/FMW infection (Figure 3C and 3D). Together, these data suggest that autophagy may not contribute to cell death or survival in NDV/FMW-infected A549 cells.

### Knockdown of autophagy-related gene *ATG5*augments NDV/FMW-mediated oncolysis in cisplatin-resistant A549 cells

**Figure 4:**
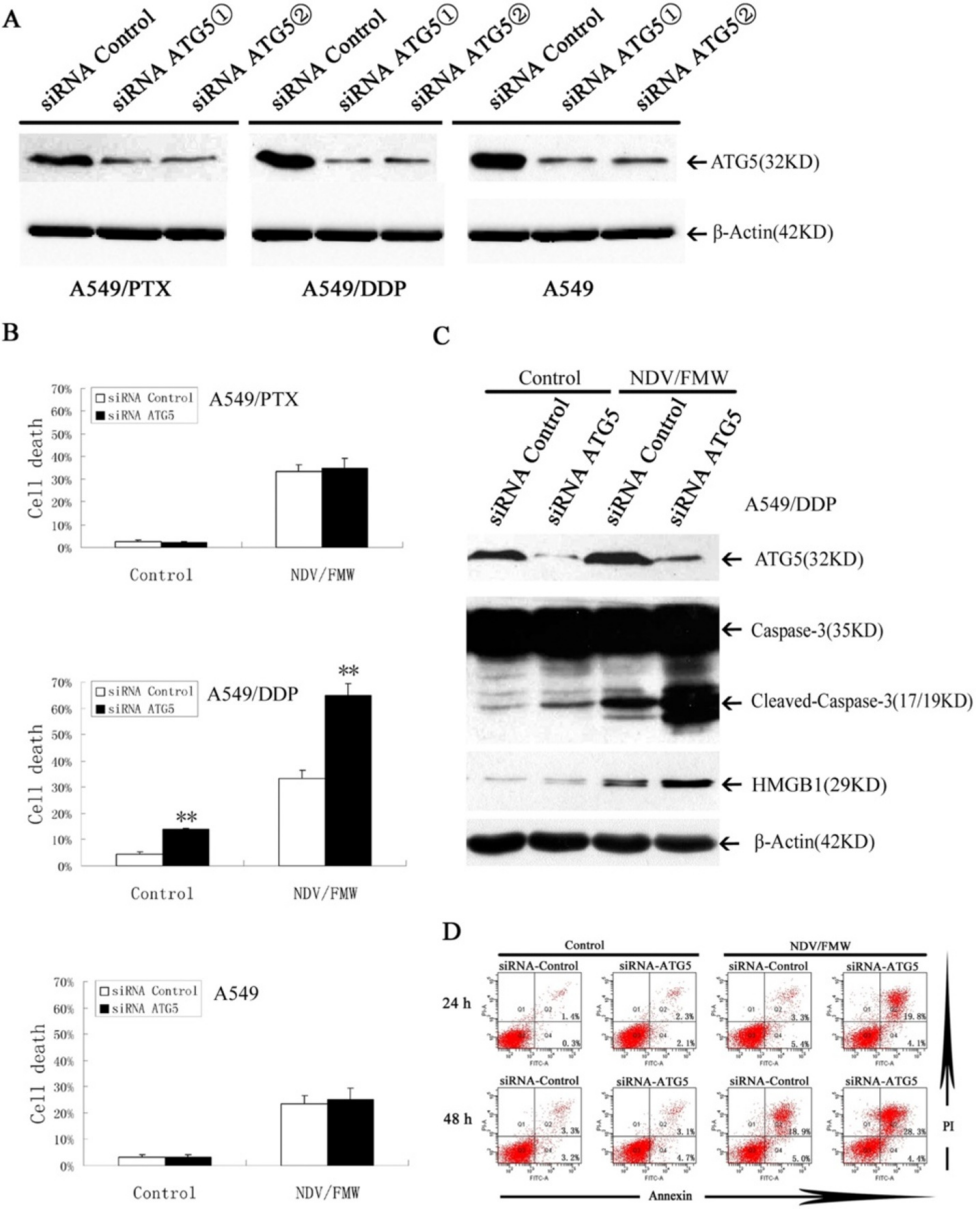
**Knockdown of ATG5 enhances NDV/FMW-mediated oncolysis in A549/DDP cells.** A549/DDP and A549/PTX as well as parental cells were transfected with either specific siRNA targeting ATG5 or scrambled siRNA. **(A)** At 72 h after transfection, the levels of ATG5 and *β*-Actin expression were measured by immunoblot analysis. **(B)** At 48 h after transfection, cells were infected with NDV/FMW (MOI =10) for 24 and 48 h respectively. Cell death was determined by MTT assay. Bar graph summarized the percentage of cell death from three independent experiments (*p < 0.05, **p < 0.01). **(C**, **D)** A549/DDP cells were transfected with either ATG5 siRNA or scrambled siRNA for 48 h, cells were then infected with NDV/FMW (MOI =10) or mock-infected for indicated times. **(C)** Cell lysates at 24 hpi was analysed by immunoblot for ATG5, cleaved caspase-3 and *β*-Actin, while cell-free supernatant was collected and determined by immunoblot for high-mobility group box1(HMGB1) **(D)**. At 24 and 48 hpi, cells were double-stained with annexin V-FITC and propidium iodide (PI), apoptosis was assessed by FACS analysis. Data shown are representative of three independent experiments.

The data shown above indicated that pharmacological modulation of autophagy enhances NDV/FMW-induced cytotoxicity. However, both rapamycin and CQ can sensitize cells towards cell death via multiple mechanisms that depend or not on autophagy. For instance, CQ can lead to apoptosis or necrosis by inducing lysosomal permeabilization [45]. To further ascertain the role of autophagy in NDV/FMW-mediated oncolysis in drug-resistant A549 cells, we knocked down expression of ATG5, which is involved in autophagosome formation, in drug-resistant A549 and parent cells by using specific siRNA targeting ATG5, and analyzed NDV/FMW-induced cell death by MTT assay. As shown in Figure 4A, cells transfected with small interfering RNAs (siRNAs) specific to ATG5 exhibited an obvious decrease of endogenous ATG5 protein. Furthermore, ATG5 knockdown significantly enhanced NDV/FMW-induced cell death in A549/DDP cells (**p < 0.01) while virus-induced cell death in A549/PTX and parent cells was not affected by ATG5 knockdown, in line with data in Figure 3B.

The execution of cell death requires an orchestrated interplay between three important processes: apoptosis, necrosis and autophagy [48, 49]. Data in Figure 3C indicated that dying cells that are double positive for PI and annexin were detected in A549/DDP cells treated with NVD/FMW or NVD/FMW with CQ at 48 hpi, suggesting that some of the cells might die via necrosis or a late necrosis consecutive to apoptosis upon virus infection and the combination treatment. To explore whether NDV-induced necrosis was modulated by regulation of autophagy, we knocked down the ATG5 protein expression using specific siRNA targeting ATG5 in A549/DDP cells. As shown in Figure 4D, at 48 hpi, markedly more dying cells that are double positive for PI and annexin were observed in ATG5-deficient A549/DDP cells than in A549/DDP cells transfected with control siRNA, suggesting that modulation of autophagy may exert an effect on NDV/FMW-induced apoptosis and necrosis. Consistent with the FACS data, we observed enhanced releasing of HMGB1 protein, a known marker of immunogenic cell death at late stages [28], in ATG5-deficient A549/DDP cells at 48 hpi compared to A549/DDP cells transfected with control siRNA (Figure 4C). We did not observe marked increase in dying cells that are double positive for PI and annexin as well as releasing of HMGB1 in ATG5-deficient A549/PTX cells upon NDV infection (data not shown). Collectively, these results suggested that ATG5 knockdown enhanced NDV/FMW-mediated oncolysis in A549/DDP cells.

### Autophagy modulation does not increase virus progeny in drug resistant cells

**Figure 5:**
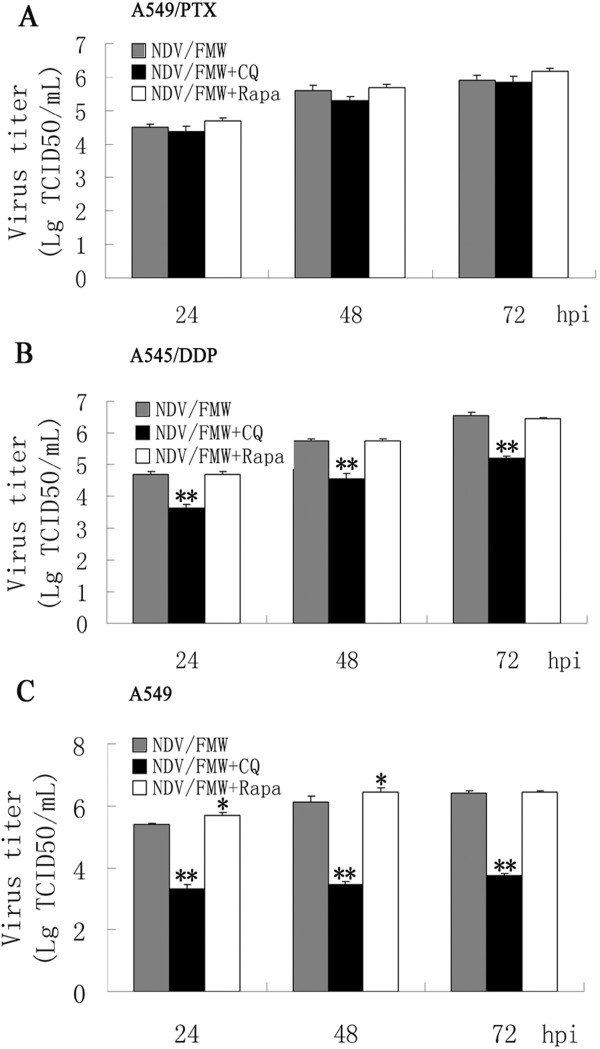
**Combination treatments do not enhance viral propagation in drug-resistant lung cancer cells.** A549/DDP and A549/PTX as well as parental cells were treated with chloroquine (CQ, 5 μM) or rapamycin (100 nM) or vehicle for 30 min. **(A-C)** Cells were then infected with NDV/FMW (MOI =0.01) for 24, 48 and 72 h respectively. Virus yield was determined at different intervals. Data are presented as the mean ± SD for triplicate assays (*p < 0.05, **p < 0.01).

To examine whether the increased oncolysis in the presence of autophagy modulators is due to the activation of apoptosis or increased viral propagation, we determined virus yield in drug-resistant A549 and parental cells treated with rapamycin or CQ. We did not observe any significant alteration in virus yield in CQ- or rapamycin-treated A549/PTX cells at the time points examined compared with control infection (Figure 5A). Interestingly, CQ treatment significantly reduced the yield of NDV/FMW progeny in A549/DDP cells at 24, 48, and 72 hpi compared with mock-treated cells (**p <0.01), while treatment with rapamycin did not alter the virus titers (Figure 5B). However, rapamycin treatment significantly increased virus yield in A549 cells, while CQ treatment resulted in a significant reduction in virus yield (Figure 5C, *p < 0.05; **p < 0.01), which is similar to our previous observations in NDV-infected U251 cells [17]. Therefore, these data suggest that the increase in viral cytotoxicity in the presence of autophagy modulators might not be due to altered viral propagation in drug resistant A549 cells.

### CQ or rapamycin potentiates NDV/FMW oncolytic activity in mice bearing drug-resistant lung cancer cells

**Figure 6:**
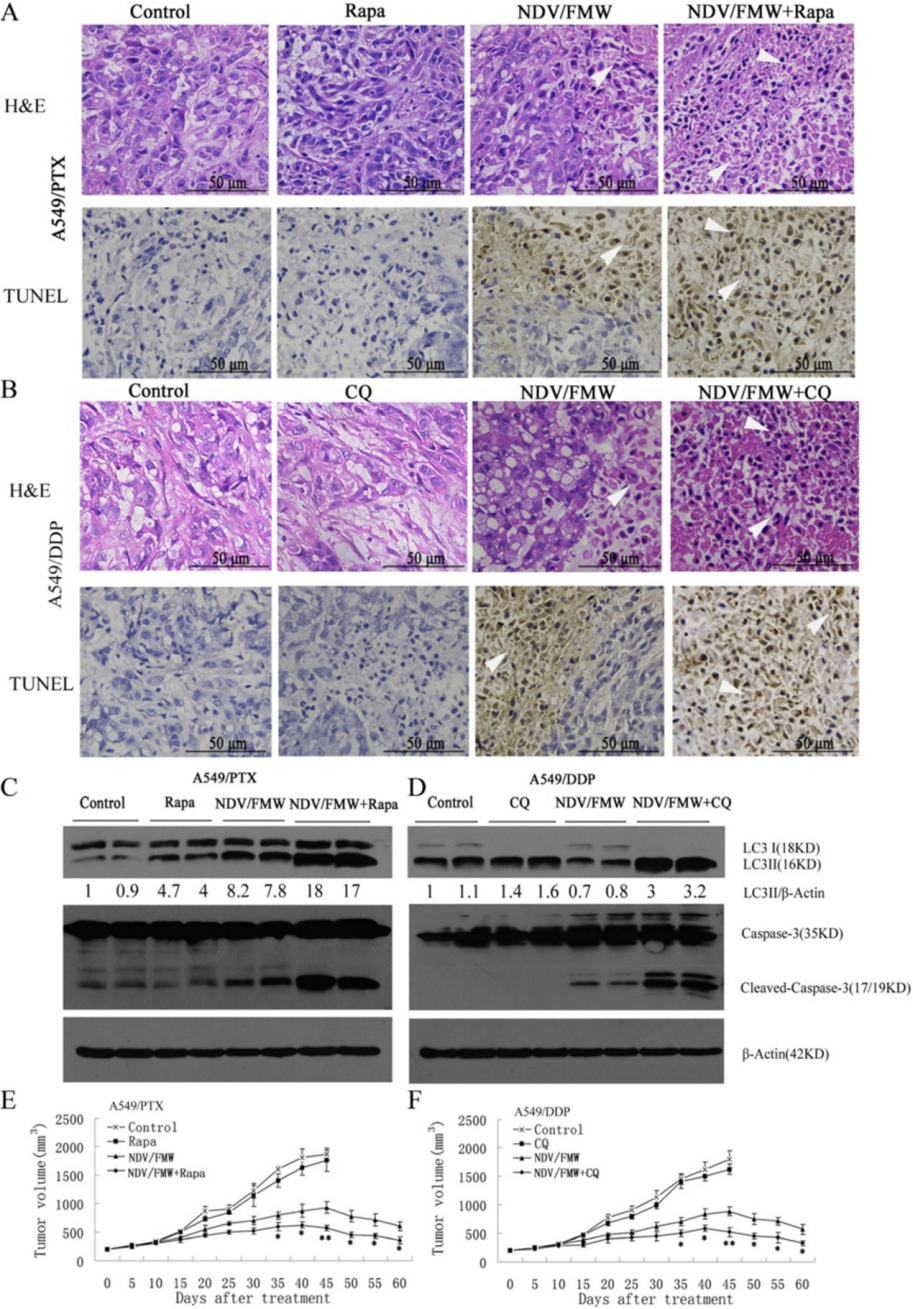
**In vivo antitumor effects of NDV/FMW enhanced by chloroquine and rapamycin.** Mice bearing A549/PTX or A549/DDP tumors were randomized into four groups: **(A)** vehicle-treated, **(B)** rapamycin (5 mg/kg) via intraperitonealy (i. p.) three times a week, or chloroquine (CQ) (45 mg/kg) alone via i.p. three times a week, **(C)** NDV/FMW (1 × 10^7^ TCID_50_ per dose) via intratumoral administration three times a week, **(D)** NDV/FMW plus CQ or rapamycin (same dose as in the above groups), CQ or rapamycin was administrated 1 d before virus injection. **(A-D)** One week after treatment, tumor tissue samples from two different animals from each treatment group (of six) were subjected to either hematoxylin–eosin (H&E) staining (The upper of A and B,tumor necrosis indicated by the arrows) or TUNEL assay (The lower of A and B, arrowheads indicate brown 3,3^'^-diaminobenzidine chromogen in cell nuclei) or immunoblot analysis of cleaved caspase-3 levels and LC3II abundance **(C, D)**. *β*-Actin was used as a loading control. **(E, F)** Mice were treated as described above for two weeks. Tumor volumes were measured at 5-day intervals for 40 days after injections and expressed as the mean ± SD (n = 10) in tumor volume–time curves. The difference in tumor regression was significant between the virus-treated and vehicle groups (*p < 0.05; **p < 0.01); group receiving the combined treatment and the single-treatment (virus alone or drugs alone) group (*p < 0.05; **p < 0.01). No statistically significant difference was observed between the groups receiving the single treatments and vehicle-treated group.

To validate the potential therapeutic use of autophagy modulators in combination with NDV/FMW, we investigated the oncolytic effects of the virus in combination with CQ or rapamycin in mice bearing A549/DDP or A549/PTX cells. The design of the *in vivo* experiments was based on previous studies from our lab and others [4, 9, 25, 26, 50, 51]. Tumor-bearing mice were intraperitoneally (i.p.) treated with vehicle, rapamycin, or CQ and were intratumorally (i.t.) administered NDV/FMW after 24 hours. To study apoptosis, tumor sections were subjected to either H&E staining or TUNEL assay. The H&E-stained tumor sections from mice treated with NDV/FMW alone or NDV/FMW in combination with CQ or rapamycin showed significant necrosis, including a loss in nuclei and cell-cell adhesion, darkly stained and condensed chromatin (Figures 6A and B upper, indicated by the arrows); in contrast, there was less tumor necrosis in tumor sections from mice treated with vehicle, CQ, or rapamycin alone (Figures 6A and B upper). TUNEL staining of tissue sections from mice bearing A549/PTX cells demonstrated that NDV/FMW in combination with rapamycin induced more apoptotic cells than NDV/FMW, rapamycin, or vehicle alone (Figure 6A lower), indicating that rapamycin enhanced the *in vivo* oncolytic efficacy of NDV/FMW in A549/PTX-derived tumor cells. Similarly, in tumor sections from mice bearing A549/DDP cells, increased numbers of apoptotic cells were observed in mice treated with NDV/FMW in combination with CQ than in mice treated with NDV/FMW, CQ, or vehicle alone (Figure 6B lower). Further analyses of caspase-3 activation in A549/PTX-derived tumors revealed that pretreatment with rapamycin led to more intense caspase-3 activation compared with the tumors that underwent NDV/FMW treatment alone (Figure 6C). Similar results were obtained for CQ-treated A549/DDP-derived tumors infected with NDV/FMW (Figure 6D). The cleaved caspase-3 levels were barely detectable in vehicle-, CQ-, or rapamycin-treated tumors. Moreover, we observed that NDV/FMW alone increased the LC3II/β-Actin ratio in A549/PTX-derived tumors compared with vehicle-treated tumors (Figure 6C), whereas it decreased the LC3II abundance in A549/DDP-derived tumors compared with the high basal level of LC3II in vehicle-treated tumors (Figure 6D). Interestingly, treatment with CQ or rapamycin alone was able to increase the LC3II/*β*-Actin ratio in these tumors, and combination treatment further strengthened this effect (Figures 6C and D).

We further investigated whether the *in vivo* combination treatments resulted in enhanced inhibition of tumor cell growth as demonstrated in our *in vitro* experiments. The treatment of tumors bearing A549/PTX cells with rapamycin alone or the addition of CQ to mice bearing A549/DDP-derived tumors had negligible therapeutic effects on tumor growth (Figures 6E and F). As expected, NDV/FMW virotherapy markedly reduced tumor growth compared with vehicle treatment (Figures 6E and F, p < 0.05, respectively). Interestingly, the combination of NDV/FMW with rapamycin induced a significant reduction in tumor volume 5 days earlier than virus alone (Figure 6E, *p < 0.05; **p < 0.01), and combination therapy also resulted in significant tumor growth inhibition compared with virus alone (Figure 6E). Similar effects were detected in mice bearing A549/DDP cells treated with NDV in combination with CQ (Figure 6F). The difference in tumor volume assessed at each time point became statistically significant at day 35 (all p values were lower than 0.05) and day 45 (all p values were lower than 0.01). Together, these data indicate that CQ and rapamycin are effective in increasing the antitumor activity of NDV/FMW in cisplatin- and paclitaxel-resistant A549 lung cancer cell mouse models.

## Discussion

Currently, the major limitation in the development of OVs in clinical trials is the low efficacy of the viruses *in vivo*. Here, we provide *in vitro* and *in vivo* evidence that pretreatment with the autophagy inhibitor CQ enhances NDV/FMW-mediated antitumor effects in A549/DDP cells via the inhibition of autophagy, while the autophagy inducer rapamycin improves the oncolytic efficacy of NDV/FMW in A549/PTX cells through enhanced autophagy, suggesting that the combined administration of autophagy modulators with oncolytic NDV/FMW may improve virotherapy in lung cancer cells resistant to various chemotherapies.

It is well known that viral infection and the cellular autophagy machinery have complex interconnections. We previously observed that NDV induces autophagy in U251 glioma cells [17]. In the current study, we showed that oncolytic NDV/FMW triggers autophagy in A549/PTX cells via the inhibition of the class I PI3K/Akt/mTOR/p70S6K pathway, which negatively regulates autophagy. However, NDV/FMW infection blocks the autophagic process in A549/DDP cells through the activation of the negative regulatory pathway. A plausible explanation for the diverse strategies that regulate the cellular autophagy machinery utilized by NDV/FMW is that the induction or inhibition of autophagy by NDV/FMW may depend on the role of autophagy in these drug-resistant lung cancer cells. Autophagy may act as a survival or cell death mechanism in drug-resistant cancers. A previous study reported that cisplatin treatment induces autophagy in A549 cells, and the acquired cisplatin resistance in A549 cells is associated with enhanced autophagy [38]. Another study suggested that cisplatin-induced autophagy might provide a prosurvival role in cisplatin-resistant SKOV3 ovarian cancer cells [52]. In line with these findings, we also detected high basal levels of autophagy in A549/DDP cells, suggesting that autophagy may act as a survival mechanism in cisplatin-resistant A549 lung cancer cells. Therefore, it is reasonable that, to induce oncolysis, oncolytic NDV/FMW should inhibit the autophagic process in A549/DDP cells. Surprisingly, the role of autophagy in paclitaxel-resistant cancer cells remains controversial. Ajabnoor *et al*. reported that an increased autophagic response was observed in paclitaxel-resistant MCF-7 breast cancer cells with reduced phosphor-mTOR and a relative resistance to the mTOR inhibitors rapamycin and RAD001 [53], suggesting that autophagy may act as a survival mechanism in paclitaxel-resistant breast cancer cells. However, Veldhoen et al. demonstrated that paclitaxel inhibits autophagy in MCF-7 and SK-BR-3 breast cancer cells [54]. Moreover, Veldhoen et al. showed that primary breast tumors that express diminished levels of autophagy-initiating genes were resistant to taxane therapy [54], suggesting that autophagy may act as a cell death mechanism in PTX-resistant breast cancer cells. In this study, we did not observe an increased basal level of autophagy in A549/PTX cells compared with parental A549 cells, indicating that autophagy may not act as a survival mechanism in A549/PTX cells. Accordingly, NDV/FMW infection resulted in the induction of autophagy in A549/PTX cells, suggesting that autophagy may play a positive role for NDV/FMW to exert its oncolytic effect in these cells. Interestingly, although NDV/FMW triggered autophagy in A549 cells, combination treatment with either rapamycin or CQ did not induce increased apoptosis, indicating that autophagy may not be involved in NDV/FMW-induced oncolysis in A549 cells.

Currently, pharmacological autophagy modulators such as rapamycin and CQ and their analogs or derivatives have been widely used in combination with OVs to enhance virotherapy for a variety of cancers in preclinical trials [26, 40–42]. However, whether autophagy inducers or inhibitors are used in combination virotherapy may be virus strain- and cancer line-dependent. Here, we presented *in vitro* evidence that rapamycin enhances NDV/FMW-mediated oncolysis in A549/PTX cells via increased autophagy, while CQ augments the antitumor effects of NDV/FMW on A549/DDP cells via inhibition of autophagosome-lysosome fusion, which is in agreement with the way in which NDV/FMW perturbs the cellular autophagy machinery in these drug-resistant lung cancer cells. The increase in NDV/FMW-mediated cytotoxicity in the presence of autophagy modulators may be due to the augmented activation of apoptosis and necrosis as demonstrated by enhanced capase-3 activation and increased numbers of apoptotic and necrotic cells. However, combination treatments may exert their effects via enhanced viral propagation. Interestingly, CQ treatment significantly reduced the yield of NDV/FMW progeny in A549/DDP cells, while pretreatment with rapamycin did not alter viral titers, suggesting that the increase in viral cytotoxicity in the presence of the autophagy inhibitor CQ might be due to enhanced activation of apoptosis and necrosis rather than altered viral replication. In contrast, pretreatment with either rapamycin or CQ did not significantly alter virus yield in NDV/FMW-infected A549/PTX cells, excluding a role for viral propagation in enhanced viral cytotoxicity by combination treatments. This notion is in agreement with studies of other OVs with combination therapies. Botta et al. reported that the inhibition of autophagy by CQ enhances the effects of the oncolytic adenovirus dl922-947 against glioma cells, while viral replication is not increased by autophagy modulation [26]. Our *in vivo* data further indicated that NDV/FMW in combination with rapamycin or CQ induces enhanced caspase-3 activation accompanied by increased LC3II abundance in A549/PTX- and A549/DDP-derived tumors. However, it should be noted that the contribution of autophagic modulators rapamycin and CQ on antitumor immunity and thus the efficacy of oncolytic virotherapy have not been taken into consideration in our *in vivo* experiments. OVs can induce autophagy and immunogenic cancer cell death which may be potentiated by co-administration with autophagy modulators as modulation of autophagy may enhance tumor immunogenecity [29]. It was reported that the combination of oncolytic adenovirus with low-dose temozolomide increased tumor cell autophagy, elicited antitumor immune responses in chemotherapy-refractory cancer patients [30]. Therefore, further *in vivo* studies are required to clarify the roles of rapamycin and CQ in antitumor immunity contributing to the overall efficacy of NDV-mediated virotherapy.

## Conclusions

In the current study, we provide evidence that pharmacological autophagy modulation enhanced the *in vitro* and *in vivo* oncolytic effects of NDV/FMW in drug-resistant lung cancer cells. Our findings suggest that the combination of NDV/FMW and autophagy modulators may be a novel treatment option for lung cancer patients with recurrent disease after cisplatin- or paclitaxel-based first-line chemotherapy. Of note, recent study demonstrated that drug-resistant NSCLC cell lines may display a stem-like signature [55, 56], linking cancer stem cell with drug resistance. Therefore, it will be interesting to extend our study to lung cancer stem cell.

## Abbreviations

CQ: Chloroquine
LC3: Microtubule-associated protein 1 light chain 3
MOI: Multiplicity of infection
ATG5: Autophagy-related gene 5
mTOR: Mammalian target of rapamycin
MTT: 3-(4, 5-dimethylthiazol-2-yl)-2, 5-diphenyltetrazolium bromide
NDV: Newcastle disease virus
NSCLC: Non-small-cell lung carcinoma
OV: oncolytic virus.

## References

[1] Brozovic A, Osmak M (2007). Activation of mitogen-activated protein kinases by cisplatin and their role in cisplatin-resistance. Cancer Lett.

[2] Hsu DS, Balakumaran BS, Acharya CR, Vlahovic V, Walters KS, Garman K, Anders C, Riedel RF, Lancaster J, Harpole D, Dressman HK, Nevins JR, Febbo PG, Potti A (2007). Pharmacogenomic strategies provide a rational approach to the treatment of cisplatin-resistant patients with advanced cancer. J Clin Oncol.

[3] Beljanski V, Hiscott J (2012). The use of oncolytic viruses to overcome lung cancer drug resistance. Curr Opin Virol.

[4] Meng S, Zhou Z, Chen F, Kong X, Liu H, Jiang K, Liu W, Hu M, Zhang X, Ding C, Wu Y (2012). Newcastle disease virus induces apoptosis in cisplatin-resistant human lung adenocarcinoma A549 cells in vitro and in vivo. Cancer Lett.

[5] Reichard KW, Lorence RM, Cascino CJ, Peeples ME, Walter RJ, Fernando MB, Reyes HM, Greager JA (1992). Newcastle disease virus selectively kills human tumor cells. J Surg Res.

[6] Schirrmacher V, Bai L, Umansky V, Yu L, Xing Y, Qian Z (2000). Newcastle disease virus activates macrophages for anti-tumor activity. Int J Oncol.

[7] Phuangsab A, Lorence RM, Reichard KW, Peeples ME, Walter RJ (2001). Newcastle disease virus therapy of human tumor xenografts: antitumor effects of local or systemic administration. Cancer Lett.

[8] Bian J, Wang K, Kong X, Liu H, Chen F, Hu M, Zhang X, Jiao X, Ge B, Wu Y, Meng S (2011). Caspase- and p38-MAPK-dependent induction of apoptosis in A549 lung cancer cells by Newcastle disease virus. Arch Virol.

[9] Wu Y, Zhang X, Wang X, Wang L, Hu S, Liu X, Meng S (2012). Apoptin enhances the oncolytic properties of Newcastle disease virus. Intervirology.

[10] Yaacov B, Eliahoo E, Lazar I, Ben-Shlomo M, Greenbaum I, Panet A, Zakay-Rones Z (2008). Selective oncolytic effect of an attenuated Newcastle disease virus (NDV-HUJ) in lung tumors. Cancer Gene Ther.

[11] Yaacov B, Lazar I, Tayeb S, Frank S, Izhar U, Lotem M, Perlman R, Ben-Yehuda D, Zakay-Rones Z, Panet A (2012). Extracellular matrix constituents interfere with Newcastle disease virus spread in solid tissue and diminish its potential oncolytic activity. J Gen Virol.

[12] Lazar I, Yaacov B, Shiloach T, Eliahoo E, Kadouri L, Lotem M, Perlman R, Zakay-Rones Z, Panet A, Ben-Yehuda D (2010). The oncolytic activity of Newcastle disease virus NDV-HUJ on chemoresistant primary melanoma cells is dependent on the proapoptotic activity of the inhibitor of apoptosis protein Livin. J Virol.

[13] Mansour M, Palese P, Zamarin D (2011). Oncolytic specificity of Newcastle disease virus is mediated by selectivity for apoptosis-resistant cells. J Virol.

[14] Szeberenyi J, Fabian Z, Torocsik B, Kiss K, Csatary LK (2003). Newcastle disease virus-induced apoptosis in PC12 pheochromocytoma cells. Am J Ther.

[15] Elankumaran S, Rockemann D, Samal SK (2006). Newcastle disease virus exerts oncolysis by both intrinsic and extrinsic caspase-dependent pathways of cell death. J Virol.

[16] Fabian Z, Csatary CM, Szeberenyi J, Csatary LK (2007). p53-independent endoplasmic reticulum stress-mediated cytotoxicity of a Newcastle disease virus strain in tumor cell lines. J Virol.

[17] Meng C, Zhou Z, Jiang K, Yu S, Jia L, Wu Y, Liu Y, Meng S, Ding C (2012). Newcastle disease virus triggers autophagy in U251 glioma cells to enhance virus replication. Arch Virol.

[18] Xie Z, Klionsky DJ (2007). Autophagosome formation: core machinery and adaptations. Nat Cell Biol.

[19] Kraft C, Martens S (2012). Mechanisms and regulation of autophagosome formation. Curr Opin Cell Biol.

[20] Kroemer G, Marino G, Levine B (2010). Autophagy and the integrated stress response. Mol Cell.

[21] Glick D, Barth S, Macleod KF (2010). Autophagy: cellular and molecular mechanisms. J Pathol.

[22] Meng S, Xu J, Wu Y, Ding C (2013). Targeting autophagy to enhance oncolytic virus-based cancer therapy. Expert Opin Biol Ther.

[23] Rodriguez-Rocha H, Gomez-Gutierrez JG, Garcia-Garcia A, Rao XM, Chen L, McMasters KM, Zhou HS (2011). Adenoviruses induce autophagy to promote virus replication and oncolysis. Virology.

[24] Jiang H, White EJ, Rios-Vicil CI, Xu J, Gomez-Manzano C, Fueyo J (2011). Human adenovirus type 5 induces cell lysis through autophagy and autophagy-triggered caspase activity. J Virol.

[25] Yokoyama T, Iwado E, Kondo Y, Aoki H, Hayashi Y, Georgescu MM, Sawaya R, Hess KR, Mills GB, Kawamura H, Hashimoto Y, Urata Y, Fujiwara T, Kondo S (2008). Autophagy-inducing agents augment the antitumor effect of telerase-selve oncolytic adenovirus OBP-405 on glioblastoma cells. Gene Ther.

[26] Botta G, Passaro C, Libertini S, Abagnale A, Barbato S, Maione AS, Hallden G, Beguinot F, Formisano P, Portella G (2012). Inhibition of autophagy enhances the effects of E1A-defective oncolytic adenovirus dl922-947 against glioma cells in vitro and in vivo. Hum Gene Ther.

[27] Cheng PH, Lian S, Zhao R, Rao XM, McMasters KM, Zhou HS (2013). Combination of autophagy inducer rapamycin and oncolytic adenovirus improves antitumor effect in cancer cells. Virol J.

[28] Bartlett DL, Liu Z, Sathaiah M, Ravindranathan R, Guo Z, He Y, Guo ZS (2013). Oncolytic viruses as therapeutic cancer vaccines. Mol Cancer.

[29] Guo ZS, Liu Z, Bartlett DL (2014). Oncolytic Immunotherapy: Dying the Right Way is a Key to Eliciting Potent Antitumor Immunity. Front Oncol.

[30] Liikanen I, Ahtiainen L, Hirvinen ML, Bramante S, Cerullo V, Nokisalmi P, Hemminki O, Diaconu I, Pesonen S, Koski A, Kangasniemi L, Pesonen SK, Oksanen M, Laasonen L, Partanen K, Joensuu T, Zhao F, Kanerva A, Hemminki A (2013). Oncolytic adenovirus with temozolomide induces autophagy and antitumor immune responses in cancer patients. Mol Ther.

[31] Jiang ZK, Johnson M, Moughon DL, Kuo J, Sato M, Wu L (2013). Rapamycin enhances adenovirus-mediated cancer imaging and therapy in pre-immunized murine hosts. PLoS One.

[32] Kim EH, Min HY, Chung HJ, Song J, Park HJ, Kim S, Lee SK (2012). Anti-proliferative activity and suppression of P-glycoprotein by (−)-antofine, a natural phenanthroindolizidine alkaloid, in paclitaxel-resistant human lung cancer cells. Food Chem Toxicol.

[33] Zou Z, Yuan Z, Zhang Q, Long Z, Chen J, Tang Z, Zhu Y, Chen S, Xu J, Yan M, Wang J, Liu Q (2012). Aurora kinase A inhibition-induced autophagy triggers drug resistance in breast cancer cells. Autophagy.

[34] Shingu T, Chumbalkar VC, Gwak HS, Fujiwara K, Kondo S, Farrell NP, Bogler O (2010). The polynuclear platinum BBR3610 induces G2/M arrest and autophagy early and apoptosis late in glioma cells. Neuro Oncol.

[35] Sun Y, Yu S, Ding N, Meng C, Meng S, Zhang S, Zhan Y, Qiu X, Tan L, Chen H, Song C, Ding C (2014). Autophagy benefits the replication of Newcastle disease virus in chicken cells and tissues. J Virol.

[36] Chen L, Meng S, Wang H, Bali P, Bai W, Li B, Atadja P, Bhalla KN, Wu J (2005). Chemical ablation of androgen receptor in prostate cancer cells by the histone deacetylase inhibitor LAQ824. Mol Cancer Ther.

[37] Kabeya Y, Mizushima N, Ueno T, Yamamoto A, Kirisako T, Noda T, Kominami E, Ohsumi Y, Yoshimori T (2000). LC3, a mammalian homologue of yeast Apg8p, is localized in autophagosome membranes after processing. EMBO J.

[38] Ren JH, He WS, Nong L, Zhu QY, Hu K, Zhang RG, Huang LL, Zhu F, Wu G (2010). Acquired cisplatin resistance in human lung adenocarcinoma cells is associated with enhanced autophagy. Cancer Biother Radiopharm.

[39] Yue Z, Jin S, Yang C, Levine AJ, Heintz N (2003). Beclin 1, an autophagy gene essential for early embryonic development, is a haploinsufficient tumor suppressor. Proc Natl Acad Sci U S A.

[40] Alonso MM, Jiang H, Yokoyama T, Xu J, Bekele NB, Lang FF, Kondo S, Gomez-Manzano C, Fueyo J (2008). Delta-24-RGD in combination with RAD001 induces enhanced anti-glioma effect via autophagic cell death. Mol Ther.

[41] Lun XQ, Jang JH, Tang N, Deng H, Head R, Bell JC, Stojdl DF, Nutt CL, Senger DL, Forsyth PA, McCart JA (2009). Efficacy of systemically administered oncolytic vaccinia virotherapy for malignant gliomas is enhanced by combination therapy with rapamycin or cyclophosphamide. Clin Cancer Res.

[42] Lun X, Alain T, Zemp FJ, Zhou H, Rahman MM, Hamilton MG, McFadden G, Bell J, Senger DL, Forsyth PA (2010). Myxoma virus virotherapy for glioma in immunocompetent animal models: optimizing administration routes and synergy with rapamycin. Cancer Res.

[43] Boya P, Gonzalez-Polo RA, Casares N, Perfettini JL, Dessen P, Larochette N, Metivier D, Meley D, Souquere S, Yoshimori T, Pierron G, Codogno P, Kroemer G (2005). Inhibition of macroautophagy triggers apoptosis. Mol Cell Biol.

[44] Amaravadi RK, Yu D, Lum JJ, Bui T, Christophorou MA, Evan GI, Thomas-Tikhonenko A, Thompson CB (2007). Autophagy inhibition enhances therapy-induced apoptosis in a Myc-induced model of lymphoma. J Clin Invest.

[45] Enzenmuller S, Gonzalez P, Debatin KM, Fulda S (2013). Chloroquine overcomes resistance of lung carcinoma cells to the dual PI3K/mTOR inhibitor PI103 by lysosome-mediated apoptosis. Anticancer Drugs.

[46] Ji C, Zhang L, Cheng Y, Patel R, Wu H, Zhang Y, Wang M, Ji S, Belani CP, Yang JM, Ren X (2014). Induction of autophagy contributes to crizotinib resistance in ALK-positive lung cancer. Cancer Biol Ther.

[47] Waqar SN, Gopalan PK, Williams K, Devarakonda S, Govindan R (2013). A phase I trial of sunitinib and rapamycin in patients with advanced non-small cell lung cancer. Chemotherapy.

[48] Chaabane W, User SD, El-Gazzah M, Jaksik R, Sajjadi E, Rzeszowska-Wolny J, Los MJ (2013). Autophagy, apoptosis, mitoptosis and necrosis: interdependence between those pathways and effects on cancer. Arch Immunol Ther Exp (Warsz).

[49] Jain MV, Paczulla AM, Klonisch T, Dimgba FN, Rao SB, Roberg K, Schweizer F, Lengerke C, Davoodpour P, Palicharla VR, Maddika S, Los M (2013). Interconnections between apoptotic, autophagic and necrotic pathways: implications for cancer therapy development. J Cell Mol Med.

[50] Alain T, Lun X, Martineau Y, Sean P, Pulendran B, Petroulakis E, Zemp FJ, Lemay CG, Roy D, Bell JC, Thomas G, Kozma SC, Forsyth PA, Costa-Mattioli M, Sonenberg N (2010). Vesicular stomatitis virus oncolysis is potentiated by impairing mTORC1-dependent type I IFN production. Proc Natl Acad Sci U S A.

[51] Thomas DL, Doty R, Tosic V, Liu J, Kranz DM, McFadden G, Macneill AL, Roy EJ (2011). Myxoma virus combined with rapamycin treatment enhances adoptive T cell therapy for murine melanoma brain tumors. Cancer Immunol Immunother.

[52] Yu H, Su J, Xu Y, Kang J, Li H, Zhang L, Yi H, Xiang X, Liu F, Sun L (2011). p62/SQSTM1 involved in cisplatin resistance in human ovarian cancer cells by clearing ubiquitinated proteins. Eur J Cancer.

[53] Ajabnoor GM, Crook T, Coley HM (2012). Paclitaxel resistance is associated with switch from apoptotic to autophagic cell death in MCF-7 breast cancer cells. Cell Death Dis.

[54] Veldhoen RA, Banman SL, Hemmerling DR, Odsen R, Simmen T, Simmonds AJ, Underhill DA, Goping IS (2013). The chemotherapeutic agent paclitaxel inhibits autophagy through two distinct mechanisms that regulate apoptosis. Oncogene.

[55] Bertolini G, Roz L, Perego P, Tortoreto M, Fontanella E, Gatti L, Pratesi G, Fabbri A, Andriani F, Tinelli S, Roz E, Caserini R, Lo Vullo S, Camerini T, Mariani L, Delia D, Calabro E, Pastorino U, Sozzi G (2009). Highly tumorigenic lung cancer CD133+ cells display stem-like features and are spared by cisplatin treatment. Proc Natl Acad Sci U S A.

[56] Barr MP, Gray SG, Hoffmann AC, Hilger RA, Thomale J, O'Flaherty JD, Fennell DA, Richard D, O'Leary JJ, O'Byrne KJ (2013). Generation and characterisation of cisplatin-resistant non-small cell lung cancer cell lines displaying a stem-like signature. PLoS One.

[CR57] The pre-publication history for this paper can be accessed here:http://www.biomedcentral.com/1471-2407/14/551/prepub

